# Seabird nutrients are assimilated by corals and enhance coral growth rates

**DOI:** 10.1038/s41598-019-41030-6

**Published:** 2019-03-12

**Authors:** Candida Savage

**Affiliations:** 1 Department of Marine Science, University of Otago, Dunedin, New Zealand; 2 Department of Biological Sciences and Marine Research Institute, University of Cape Town, Cape Town, South Africa

## Abstract

Nutrient subsidies across ecotone boundaries can enhance productivity in the recipient ecosystem, especially if the nutrients are transferred from a nutrient rich to an oligotrophic ecosystem. This study demonstrates that seabird nutrients from islands are assimilated by endosymbionts in corals on fringing reefs and enhance growth of a dominant reef-building species, *Acropora formosa*. Nitrogen stable isotope ratios (δ^15^N) of zooxanthellae were enriched in corals near seabird colonies and decreased linearly with distance from land, suggesting that ornithogenic nutrients were assimilated in corals. In a one-year reciprocal transplant experiment, *A. formosa* fragments grew up to four times faster near the seabird site than conspecifics grown without the influence of seabird nutrients. The corals influenced by elevated ornithogenic nutrients were located within a marine protected area with abundant herbivorous fish populations, which kept nuisance macroalgae to negligible levels despite high nutrient concentrations. In this pristine setting, seabird nutrients provide a beneficial nutrient subsidy that increases growth of the ecologically important branching corals. The findings highlight the importance of catchment–to–reef management, not only for ameliorating negative impacts from land but also to maintain beneficial nutrient subsidies, in this case seabird guano.

## Introduction

Nutrient subsidies can transcend ecosystem boundaries where they can enhance productivity^1^ and functional diversity^2^, alter food webs^3^, and increase stability^4^ and persistence of recipient marine communities^5^. Allochthonous nutrients can transcend ecotones either passively, such as macroalgal detritus that washes up on coastlines^3,6,7^, or via active vectors including seabirds^1,8^. The ecological effects of these nutrient subsidies are particularly pronounced when the receiving ecosystem has low production^5,9^. A case in point is the Gulf of California islands where seabirds forage in highly productive marine waters and deposit guano around their roosting sites that enhance local productivity^1^ and influence community structure in terrestrial desert ecosystems^4,10^. Nutrient enrichment from seabird colonies can also increase marine production via sea–land–sea transfer. For example, ornithogenic nutrients increased macroalgal production^11^ and altered benthic community structure of a temperate intertidal rocky reef community^12^. In tropical ecosystems, seabird nutrients can enrich nitrogen inputs to soil on islands^8,13^ and increase nutrient availability in adjacent pelagic^14^ and benthic food webs^15^. Seabird-derived nutrients have been traced into coral holobionts^16^, however the ecological effects of these nutrients on reef-building (scleractinian) corals have not been demonstrated previously. This study assessed the influence of seabird nutrient subsidies on coral growth rates using a spatial gradient sampling scheme and a reciprocal transplant experiment.

Coral reefs are among the most productive ecosystems yet occur in oligotrophic waters^17^. This paradigm is due largely to the tight coupling in nutrient cycling between the coral host and endosymbionts (commonly referred to as zooxanthellae), whereby inorganic nutrients excreted by the coral animal are assimilated by the symbiotic dinoflagellates of the family Symbiodiniaceae^18^ to support photosynthesis^19^. In turn, the zooxanthellae translocate organic compounds to the coral animal to support metabolic demands^19^. Within the coral holobiont, endosymbionts can acquire inorganic nutrients from their host’s waste metabolites or from surrounding seawater^20^. At a community level, the mutualistic association between the branching coral *Sylophora pistillata* and the coral obligate damselfish *Dascyllus marginatus* results in significantly higher growth rates of corals with resident damselfish due to nutrient subsidies from the fish waste^21^. Thus, external nutrients that elevate local nitrogen conditions in waters surrounding corals can increase zooxanthellae density, enhancing photosynthesis and coral growth rates^22,23^. However, there are environmental constraints and energetic costs associated with the maintenance of the mutualistic association between corals and endosymbionts with some studies showing that excessive nutrients can act like a stressor and cause a breakdown in the coral-algal symbiosis^24^.

Elevated nutrient concentrations to coral reefs today are typically associated with anthropogenic sources including human sewage^25–28^ and agricultural fertilizer^29,30^, where their effects are often considered detrimental to the coral reef ecosystem^31^. By contrast, nutrient subsidies from natural nutrient sources such as bird guano are principally excreted in an organic form of nitrogen^32^ that undergoes speciation into various forms of nitrogen^33^ and it remains to be shown whether it acts as a natural analogue to anthropogenic nutrient inputs. Nutrients generally increase cell densities of endosymbionts^22^, however the biochemical effect of this on corals is conflicting. Some studies show an increase in photosynthetic performance^34^ and calcification^35,36^ with increased nutritional supply. Conversely, other studies show a decrease in autotrophy caused by a chemical imbalance in the zooxanthellae^37^ and a build-up of reactive oxygen species^38,39^ which affects the stress tolerance of corals^40^. The relationship between nutrient availability and coral growth and photobiology is context-dependent, with exogenous factors like nutrient source likely a key determinant of the direction of the response at an individual coral level^41^. At the community level, excess nutrients can alter coral reproduction^42^ and lead to loss of coral diversity and percent cover^43^. It can stimulate macroalgal growth and give algae a competitive advantage over slower-growing reef-building corals that once established, can create changes in chemical conditions on the reef^44,45^ that maintain the reef in a macroalgal dominated state^46^. However, most studies on nutrient impacts on corals have been conducted on reefs that are already in a degraded state^47^ or subject to multiple stressors in addition to excess nutrient availability^48^, including habitat transformation^49^ and overfishing^50^. The reduction in numbers of herbivorous fishes, even at low levels of subsistence fishing^51^, together with increased nutrient delivery has been shown to erode resilience of coral reefs and cause transitions from healthy coral-dominated reefs to degraded algal-dominated systems^52^. By contrast, there are few studies on the effects of nutrient subsidies to coral reefs in less-disturbed ecosystems^14,16,53^, and no studies that have investigated the effects of seabird nutrients on coral growth rates.

**Figure 1:**
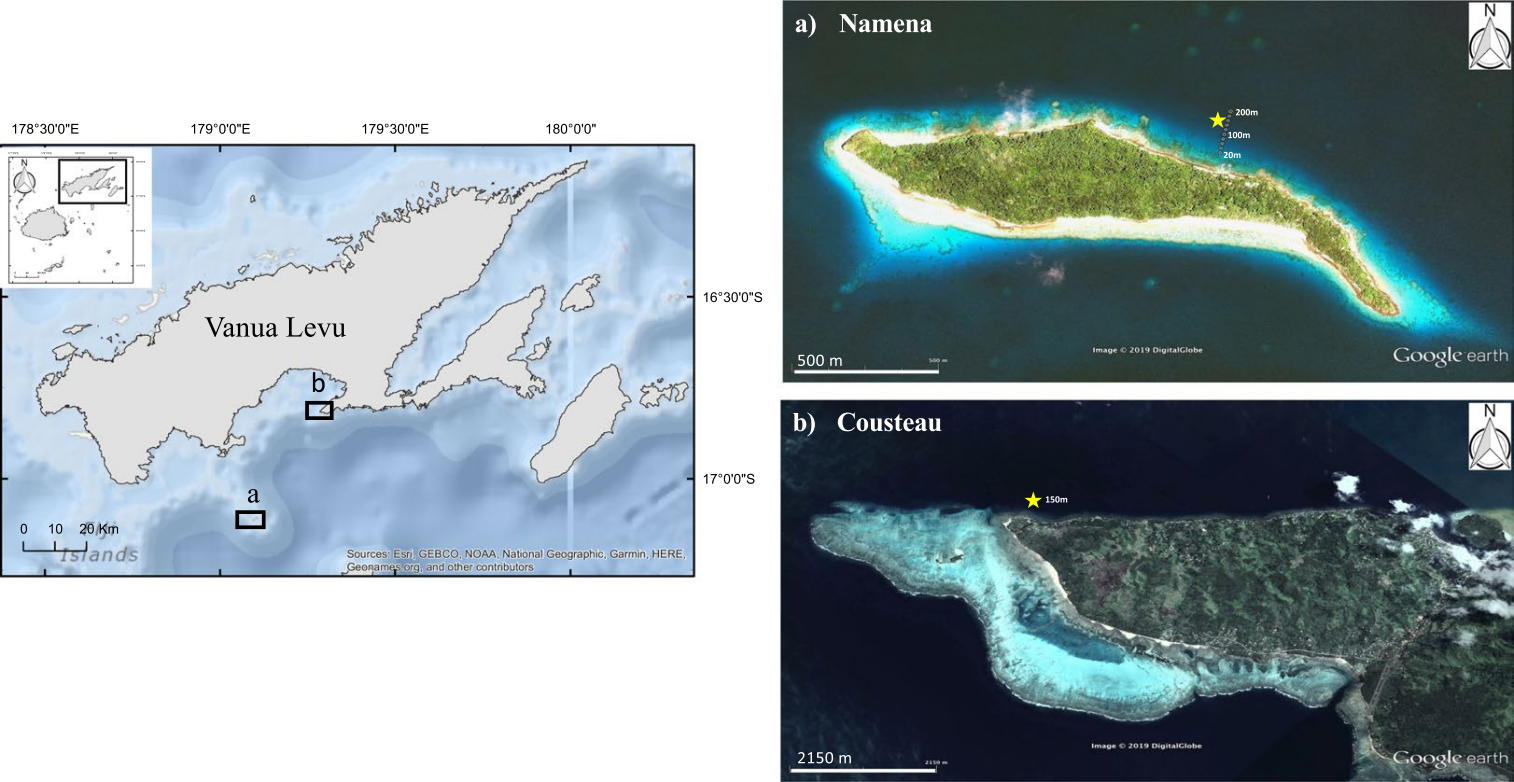
Location of study sites. Left: The Fiji archipelago (insert) and the position of the northern division, Vanua Levu, where the study sites are located. Right: (**a**) Namena island, with abundant populations of breeding seabirds, and (**b**) Cousteau on Vanua Levu. The spatial transect sites are shown as circles and the reciprocal transplant sites as stars. Map of Fiji and Vanua Levu created using Geographic Information System ArcGIS v.10.2 and the satellite images were obtained from Google Earth v.7.3.2.

This study assessed whether seabird-derived nutrients assimilated by corals enhances coral growth rates. To investigate the spatial influence of seabird nutrients on one of the dominant reef-building corals in the Pacific, *in hospite* colonies of *Acropora formosa* were sampled every 20 m (from 20 m to 200 m) perpendicular to shore from Namenalailai (hereafter Namena), a remote island with abundant nesting seabirds and a large marine protected area. The zooxanthellae were extracted from these coral samples and analyzed for cell density and natural abundance stable isotope ratios of nitrogen (δ^15^N). Since ornithogenic nitrogen is enriched in ^15^N over background levels of nutrients^8,15,54–56^, δ^15^N provides a natural tracer that can be used to assess the influence of seabird-derived nutrients in corals^16^. We expected a decreasing trend in δ^15^N values and zooxanthellae densities in corals with increasing distance from the island consistent with a decreasing influence of seabird nutrient subsidies. To test whether seabird nutrients enhance growth rates of coral, we used a reciprocal transplant experiment for one year with fragments of *A. formosa* between Namena and Cousteau, the closest practical site with a similar physical environment but without nesting seabirds (Fig. 1). Cousteau is located on the island of Vanua Levu, it is also a marine protected area and had a few colonies of *A. formosa* at ca. 150 m offshore that were suitable for the transplant experiment. We hypothesized that growth rates of corals near the seabird roosting island would be greater than conspecifics from reefs without seabird colonies due to elevated nutrient availability from seabird guano.

## Results

### Nutrient characteristics of the two sites

Dissolved inorganic nitrogen (DIN) concentrations were significantly elevated in the waters of the nearshore coral reef at Namena with DIN concentrations up to 12.7 µM compared to 1.8 µM at Cousteau (Table 1). Concentrations of nitrate (Wilcoxon W = 20, p = 0.025) and ammonia (Wilcoxon W = 21, p = 0.031) were significantly elevated at Namena relative to Cousteau. There was temporal variation in nutrient concentrations, with extremely high nitrate concentrations (up to 11.5 µM) measured in April 2013 at Namena. Phosphate concentrations also tended to be higher in April 2013 at both sites, although this was not significant. Phosphate concentrations were not statistically different (p > 0.05) between the transplant sites. The N:P in seawater was higher at Namena, with a ratio between 14–33 compared to Cousteau at 3–5.

**Table 1:** Nutrient concentrations (mean ± S.E.) in the water column at the seabird-influenced marine protected area (MPA) site, Namena, and another MPA without seabirds, Cousteau.

Site	Sampling (Month, Year)	n	Ammonia (μM)	Nitrate (μM)	DIN (μM)	Phosphate (μM)	N:P ratio
Namena	Before transplant (December, 2011)	5	1.202 ± 0.256	0.529 ± 0.289	1.73	0.125 ± 0.071	13.83
	After transplant (April, 2013)	5	1.172 ± 0.416	11.543 ± 0.416	12.72	0.391 ± 0.110	32.49
Cousteau	Before transplant (December, 2011)	5	0.33 ± 0.11	0.01 ± 0.00	0.33	0.11 ± 0.02	2.93
	After transplant (April, 2013)	5	1.06 ± 0.32	0.73 ± 0.14	1.79	0.439 ± 0.12	4.54

Samples (n = 5 per sampling occasion) were taken mid-water (~2 m) above the coral transplant arrays before the spatial transect sampling and the initiation of the transplant experiment (December 2011), and after the reciprocal transplant experiment (April 2013).

### Spatial gradient of endosymbiont parameters at Namena

**Figure 2:**
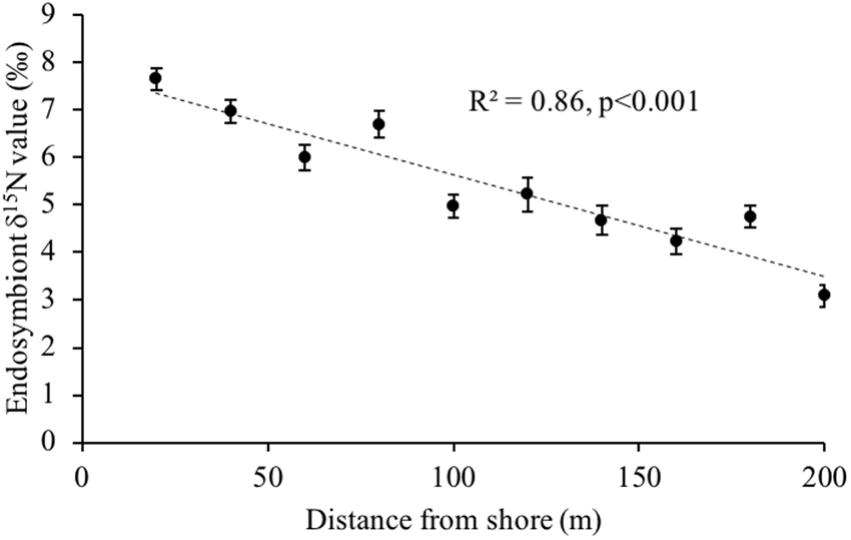
Spatial transect. The stable nitrogen isotope values, δ^15^N, of extracted endosymbionts with distance from shore (in meters) on the leeward side of Namena island, Fiji. Values are mean ± 1 S.E. (n = 9) for the three *Acropora formosa* colonies sampled at 20 m intervals along the three transect lines perpendicular to the shore. Each colony is a pooled and homogenized sample of 3–5 fragments. Dashed line represents the linear regression, R^2^ = 0.86, p < 0.001.

**Figure 3:**
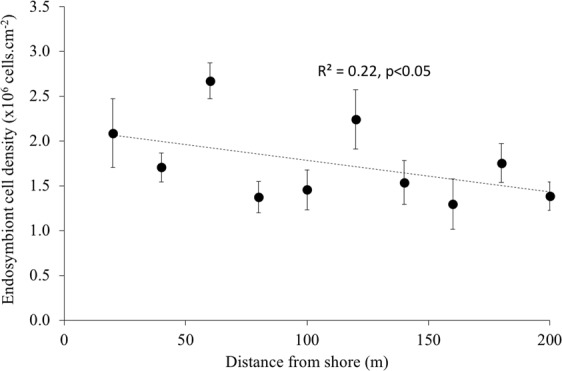
Spatial transect. Cell density of endosymbionts (x10^6^ cells per cm^−2^ host tissue) with distance from shore (in meters) on the leeward side of Namena island, Fiji. Values are mean ± 1 S.E. (n = 9) for the three *Acropora formosa* colonies at 20 m intervals along the three transect lines perpendicular to the shore. Each colony is a pooled and homogenized sample of 3–5 fragments. Dashed line represents the linear regression, R^2^ = 0.22, p = 0.016.

The δ^15^N values of extracted zooxanthellae decreased significantly with distance from land at Namena (F_2,28_ = 177.4, p < 0.001) with an R^2^ of 0.86 (Fig. 2). The mean δ^15^N value for endosymbionts decreased from 7.7‰ at 20 m to 3.1‰ at 200 m from the island. Similarly, symbiont density was greater in coral colonies closer to land and decreased significantly with distance from shore at Namena (F_2,28_ = 6.639, p = 0.016), although the relationship was weak (Fig. 3). When considering all sampled corals growing naturally within 200 m of the seabird roosting site at Namena island, the average density of zooxanthellae cells in corals was 1.7 × 10^6^ cells.cm^−2^ host tissue (n = 30).

### Reciprocal transplant experiment

**Figure 4:**
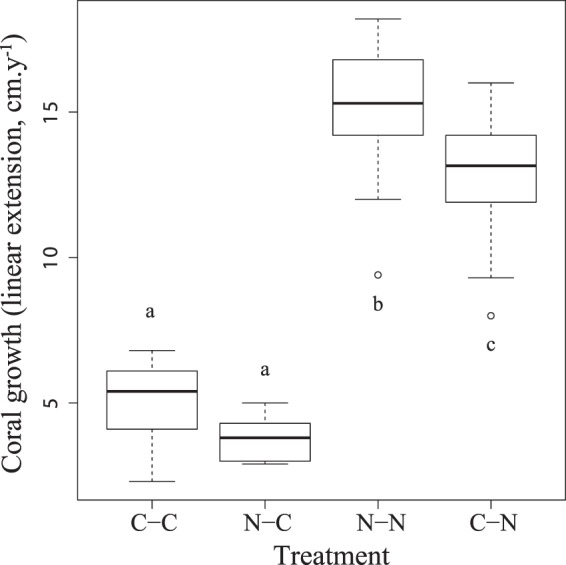
Transplant experiment. Coral growth (linear extension in cm.y^−1^) of *Acropora formosa* fragments from the one-year reciprocal transplant experiment between Cousteau (C) and Namena (N), with the median and interquartile range shown in box-and-whisker plots. Treatments (left to right): C–C = fragments from Cousteau and retained at their natal site; N–C = fragments from Namena and transplanted to Cousteau; N–N = fragments from Namena and retained at their natal site; C–N fragments from Cousteau and transplanted to Namena. Significantly different treatments according to Tukey’s Post Hoc tests are denoted by letters.

**Figure 5:**
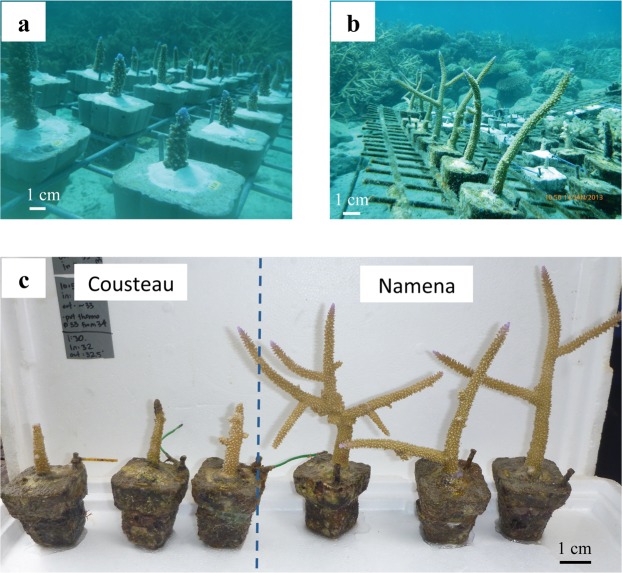
Transplant experiment. (**a**) Individually-labelled fragments of *Acropora formosa* grown at Namena when the nubbins were created in January 2012, and (**b**) one year later in January 2013. (**c**) Examples of *A. formosa* fragments that originate from Namena and were transplanted to Cousteau (N–C: three nubbins on left) or retained at Namena (N–N: three fragments on right) after one year. Photographs: C. Savage.

Coral growth rates (measured as skeletal linear extension) were significantly different between coral nubbins grown at Namena and Cousteau (F_3,68_ = 210.6, p < 0.001), with fragments maintained at Namena exhibiting up to four times greater linear extension rates than conspecifics transplanted to Cousteau (Figs 4, 5). Tukey’s post-hoc tests showed that the corals from Namena that were maintained at their natal site (N–N) achieved significantly higher growth rates (mean 15.29 ± 0.35 cm.y^−1^) than other nubbins. The next highest growth rates (mean 12.79 ± 0.33 cm.y^−1^) were fragments from Cousteau that were transplanted to Namena (C–N) for one year. By contrast, fragments outplanted at Cousteau that were collected from Cousteau (C–C: mean 5.08 ± 0.27 cm.y^−1^) or Namena (N–C mean 3.75 ± 0.20 cm.y^−1^) had significantly lower growth rates. There was no mortality during this experiment.

The water temperature averaged 28.1 °C at Cousteau (range: 23.3–30.7 °C) and 27.8 °C at Namena (range: 23.2–32.01 °C) during the four months for which data were reliably recorded with loggers. There was no significant difference in the average monthly temperature between the transplant sites (Welch Two-Sample t-test, t_1,6_ = 0.482, p = 0.647). The average light environment at Namena tended to have slightly higher incident light (average PAR: 613 µmol photons m^−2^ s^−1^) compared to Cousteau (average PAR: 568 µmol photons m^−2^ s^−1^), however this was not significantly different (Welch Two-Sample t-test, t_1,6_ = −0.220, p = 0.834).

## Discussion

This is the first study to demonstrate a positive effect of seabird nutrient subsidies for corals, with significantly greater growth rates of a dominant branching coral near a seabird island. Elevated nutrients delivered to nearshore coral reefs adjacent to a breeding colony of seabirds provided a bottom-up nutrient subsidy that was assimilated by endosymbionts, as reflected by decreasing δ^15^N values of zooxanthellae with distance from shore. *Acropora formosa* colonies growing in proximity to this elevated nutrient source and fragments transplanted from distant reefs to the area exhibited growth rates four times greater than conspecifics grown at the same depth on a coral reef without seabird-derived nutrients. Therefore, in contrast to excess anthropogenic nutrients, seabird guano can benefit coral reefs, which should be considered in catchment–to–reef management, particularly given the worldwide threat to seabirds.

### Seabird nutrients elevate nitrogen availability

Nutrients were significantly elevated in seawater bathing the fringing reefs on the leeward side of Namena island, Fiji, where seabirds including 1000–3000 breeding pairs of red-footed boobies (*Sula sula*) roost year round^57^. The gradient of decreasing δ^15^N values in extracted endosymbionts with distance from shore indicated that the elevated nitrogen source was most likely ornithogenic, since there are no rivers or point sources of nutrients on the island and seabird guano is enriched in ^15^N relative to background nitrate δ^15^N values^8,15,54,56,58^. Guano δ^15^N values are >10‰ for seabirds^59^, with red-footed booby guano reported as 11‰^16^ and decaying guano on another Fijian seabird island having δ^15^N values as high as 50‰^8^. Local nitrate enrichment and elevated δ^15^N values in corals have been linked with nesting seabirds, where ornithogenic nutrients can contribute 15–50% of the nitrogen requirements of the coral *Pocillopora damicornis*^16^. Thus, the findings of this study are consistent with seabird nutrients elevating nitrogen availability on local reefs and being assimilated by the endosymbionts. However previous studies have not assessed whether an ornithogenic nutrient subsidy has a direct effect on coral growth.

This study shows that reef-building corals grown near a large seabird colony exhibited growth rates up to four times greater than conspecifics from the same area that were transplanted distant from seabird nutrients. Linear extension rates of 15 cm.y^−1^ at Namena are amongst the highest rates reported in the literature for comparable growth experiments of *Acropora* fragments^60–62^. The light conditions were above saturation levels for corals^63^ and since there were no significant differences in the light or temperature conditions between the transplant sites and wave energy was similar with both sites north-facing and sheltered from the prevailing south-east trade winds, it suggests that inter-site differences in growth were mainly driven by the different nutrient conditions.

Seabird guano elevates dissolved organic nitrogen^32^, inorganic nitrogen^15,16^ and phosphate concentrations in seawater^15,58^. In this study, there was no significant difference in measured phosphate concentrations between Namena and Cousteau, despite differences in seabird populations. Phosphate fluxes may have been higher from seabird guano, however if this is assimilated readily by benthic organisms it would not show in the water column concentrations. Nevertheless, phosphate concentrations were elevated and not limiting at both sites^64,65^, suggesting that the endosymbionts were replete in phosphorus to support coral growth and metabolism^66^. Nitrogen concentrations in the water column, in comparison, were significantly different between sites with ammonia and nitrate significantly elevated at the seabird site (Namena) compared to Cousteau and other coastal sites in Fiji^64^. It should be cautioned that nutrients are temporally variable and this study reports on only two sampling occasions, however nitrate concentrations were significantly elevated for both sampling intervals at the seabird site. Large bird populations on small islands can result in extremely high nitrate concentrations in groundwater, which is advected into adjacent coastal lagoons^33^. In this study, despite nitrate concentrations above thresholds considered harmful to corals^64,65^, the *A. formosa* fragments growing near the seabird nesting island remained healthy during the experiment, grew vigorously and had endosymbiont cell densities considered optimal^67^ for branching corals^68–70^ to maintain photosynthetic performance^71^. The findings provide an interesting perspective on the contested issue of whether excess nutrients are harmful or beneficial to coral reefs^31^.

The findings in this study suggest that natural sources of nutrient enrichment to the coast like seabird guano can have positive effects on acroporid corals in contrast to anthropogenic nutrient sources^41^. Guano nutrient subsidies have increased production of mangroves^72^ and seagrass^73^ and the current study shows that ornithogenic nutrients result in a nutrient-replete environment that can enhance coral production. The composition of seabird guano contains essential nutrients (nitrogen, phosphorus), including trace elements^58^ and iron^74^, in sufficient amounts that biochemical functions remain stable^37^. Changes in nutrient stoichiometry can affect carbon acquisition and nutrient partitioning in the coral holobiont^37,75^. The seawater near the seabird colony had N:P which approximated Redfield ratio^76^ in contrast to the site distant from seabirds. Thus the stoichiometric balance and nutrient source^41^ is also important to consider along with input rates in determining the effect of nutrients on coral performance and production.

An important caveat is that the reef where the study was done is located within a no-take marine protected area with abundant fish populations^77,78^. There are numerous studies that have documented phase shifts in benthic community composition from scleractinian corals to a degraded macroalgal-dominated state^46,79^ following nutrient enrichment^26,80^, particularly with declines in herbivorous fishes^81–83^. In this study, the elevated nutrients from the seabirds didn’t promote nuisance macroalgal blooms^84^ despite the highly elevated DIN concentrations, most likely due to the presence of healthy fish populations, which would have maintained critical ecosystem functions like grazing and bioerosion^53^ that prevents establishment of macroalgae.

### Conservation and management implications

Marine conservation tends to focus on connectivity among reefs within a seascape to inform management decisions including where to locate marine protected areas^85^. However, catchment-to-reef connectivity can also be important to consider in marine management and conservation^86,87^, not only for taking into account the negative consequences from land, for example increased sediment inputs^29^, but also for positive gains when coral reefs are adjacent to pristine forested landscapes^14^. As shown in this study and other recent papers^14–16,53^, seabirds can provide important nutrient subsidies to the adjacent coast where seabird roosting sites are adjacent to coral reefs. Given that nearly one-third of seabird species are at risk of extinction globally^88^, conservation needs to consider possible effects of declines in this nutrient subsidy on coral growth around pristine remote atolls and reefs. To this end, Namena may provide an ideal before-and-after study system to investigate the effects of a decline or loss of guano for the adjacent coastal ecosystem as the island experienced severe destruction from hurricane Winston in February 2016 after this study was conducted and most seabird roosting sites were destroyed (pers. obs.). Apart from the direct effects of storm damage to the fringing coral reefs, investigating the indirect effects of a severe reduction in ornithogenic nutrients would advance our understanding of the role of allochthonous nutrient subsidies on productivity and recovery following disturbance.

## Methods

### Study site

Namena is a ~0.5 km^2^ island within the Kubulau District in northern Fiji that provides a model ecosystem to investigate the role of ornithogenic nutrient subsidies on coral growth without the confounding effects of other human stressors. Namena Marine Reserve is the largest (60.6 km^2^) and oldest (established 1997) no-take marine protected area in Fiji^78^ with high coral cover and abundant fish populations including healthy populations of top predators^77^. Namena’s marine reserve is strictly no-take and compliance is self enforced by the local communities^78^. The island has an intact coastal forest with abundant populations of roosting seabirds, including an estimated 1000–3000 breeding pairs of red-footed boobies, *Sula sula* (population estimate: 1986–2008)^57^. The closest practical site without nesting seabirds for the transplant experiment is adjacent to the Cousteau resort on the island of Vanua Levu. While Cousteau had lower live coral cover than Namena that prevented comparative sampling along a spatial gradient every 20 m from shore, the focal species *A. formosa* was found ca. 150 m offshore, which enabled fragmentation to create transplant nubbins. Cousteau is a no-fishing marine protected area since 2000 and was extended in area in 2005. The physical environment is similar between Cousteau and Namena with comparable depth where the transplant corals were collected, water temperature and wave energy were similar and both sites were north-facing, thus protected from the prevailing south-east trade winds.

### Spatial transect sampling

At Namena, samples of *Acropora formosa* colonies were collected for analyses of zooxanthellae density and nitrogen isotope ratios (δ^15^N) in January 2012. Transect lines were conducted perpendicular to the shore and sampling done at 20 m intervals between 20 m from land to 200 m seaward. Fragments (ca. 5 cm) of *A. formosa* were collected from attached colonies by snorkeling along the transect line and collecting fragments at a depth of approximately 3 m. If *A. formosa* colonies were not available on the transect line, another colony within a 1.5 m radius of the transect line at the same distance from shore was sampled. Three transects were taken perpendicular to land approximately 50 m apart, and at each 20 m increment three separate *A. formosa* colonies were sampled by collecting between 3–5 fragments per colony (depending on availability). These fragments collected from a single colony were pooled and homogenized to get an averaged δ^15^N value and zooxanthellae count per colony. The coral samples were immediately frozen and processed individually in the laboratory for endosymbiont density and stable isotope ratios. In total, nine samples were analyzed at each 20 m distance. These samples were collected with an approved permit (Fiji Immigration Research Permit 3273/11).

### Transplant experiment

A reciprocal transplant experiment was conducted between Namena and Cousteau. Coral fragments of *A. formosa* were created from visually healthy colonies at Namena and Cousteau between 08–16 January 2012 using established procedures^89^. The initial sizes of the fragments were comparable at the two sites, ranging between 3 cm and 10 cm, with fragment size determined by the size and shape of the colony from where they were collected. The nubbins were placed on individual, labeled (Hallprint®) concrete blocks using underwater epoxy and measured using calipers. They were left in aerated tanks under shade cloth for ca. 2 hours to establish on the bases before being planted out *in situ*. A total of 36 coral fragments were created at each site with half (n = 18) being retained at the natal site and half (n = 18) transplanted to the other site. Samples were transported in large containers with site seawater, under shade cloth and using battery-operated air bubblers to minimize stress during the 1-hour boat transport time between sites. At each site, the coral fragments were placed on a customized array at 3 m depth and elevated 50-cm off the seabed. The arrays were located ca. 150 m from land at both sites, on the leeward side of Namena Island, Fiji (17°6′26.66″S, 179°6′6.21″E) and at Cousteau (16°48′43.57″S, 179°17′11.59″E). These sites were chosen to be sufficiently close to land to be influenced by land-derived nutrient sources but deep enough to prevent wave damage or interference from snorkelers. The coral fragments were left to grow for 12 months, after which time the individual fragments were collected and measured to quantify growth using calipers. Growth was recorded as skeletal linear extension, including growth of side branches as well as the main axial branch of each fragment^60^.

Samples of water column nutrient concentrations were collected mid-water (~2 m) above the transplant arrays at both transplant sites. The nutrient samples were taken in December 2011, 3 weeks before the spatial transect sampling at Namena and the initiation of the transplant experiment, and again in April 2013, after the reciprocal transplant experiment. The seawater samples were taken in acid-washed vials and immediately filtered through pre-combusted Whatman 0.45 μm GFF filters and stored on ice until frozen (within 2 h) at −20 °C. Samples were analyzed within 2 months of collection for dissolved inorganic ammonia (NH_4_^+^), nitrite/nitrate (NO_2_^−^/NO_3_^−^), and phosphorus (PO_4_^2+^) concentrations on a Lachat QuikChem 8500 series 2 Flow Injection Analysis autoanalyser.

HOBO^®^ pendant temperature/light 64k data loggers (Onset) were deployed at the two transplant sites to measure the temperature and light environment at each of the arrays. Two loggers were attached on diagonally opposite corners of each array at the height of the coral fragments and set to log at 10-minute intervals. The HOBO light loggers record in Lux and were therefore calibrated by simultaneous recording underwater using a cosine corrected LI-COR^®^ underwater sensor (LI-192 underwater quantum sensor coupled with a LI-250A light meter, LI-COR) and the data reported in PAR (µmol photons m^−2^ s^−1^) using a correction following established methods^90^.

### Laboratory analyses

The zooxanthellae were extracted from the coral fragments using a waterpik^91^ and 0.2 µm filtered site seawater. Zooxanthellae were separated from animal tissues using four centrifugation steps (2700 g for 10 min). The pellet containing the zooxanthellae was resuspended in 10 mL of 0.2 µm filtered sterile seawater and a known volume filtered onto pre-combusted GF/F 0.45 µm filters and dried for stable isotope analyses. The filters were analyzed for nitrogen stable isotope ratios at Isotrace, Department of Chemistry, University of Otago, on a Europa Hydra mass spectrometer coupled to a Carlo Erba NC 2500 elemental analyser. The isotope ratios are reported in the delta notation:

$${{\rm{\delta }}}^{{\rm{15}}}{\rm{N}}=[({{\rm{R}}}_{{\rm{sample}}}/{{\rm{R}}}_{{\rm{standard}}})\,-{\rm{1}}]\times {\rm{1000}}$$

where R refers to the ratio ^15^N:^14^N and all values are reported in the units, per mil (‰). Raw isotope ratios are normalized by three-point calibration to the international scales using two IAEA (International Atomic Energy Agency) reference materials (USGS-40 and USGS-41) and a laboratory standard (EDTA-OAS, Elemental Microanalysis Ltd, UK). EDTA-OAS has multi-year and multi-laboratory calibration records against IAEA reference materials and is used as a drift control material by assaying a pair of aliquots after every twelve samples of a batch. Precision for δ^15^N is ± 0.2‰.

A second aliquot of the resuspended pellet was used to determine cell density. The cell density of endosymbionts was counted using a Scepter 2.0 handheld automated cell counter (Millipore) with a 40 µm sensor after diluting the extracted zooxanthellae samples 2:1 in phosphate-buffered saline (PBS) and checking for accuracy on select samples using a haemocytometer. The surface area of the coral fragment used was measured according to the paraffin wax dipping technique^92,93^ and the symbiont density normalized as cells.cm^−2^ host tissue.

### Statistical analyses

The isotope (δ^15^N) and zooxanthellae density (cells.cm^−2^) data for the three replicate colonies along each transect line with distance from shore were averaged and analyzed using Generalised Linear Models (GLM) with distance from land a fixed factor and the measured symbiont parameters analyzed as continuous predictor variables.

Growth of the coral fragments from the reciprocal transplant experiment were compared after testing for normality and homoscedasticity of variances using a one-way analysis of variance (ANOVA) and Tukey’s Post Hoc tests.

To test for differences in nutrient concentrations between the Namena and Cousteau transplant sites the nutrient concentrations (ammonia, nitrate, phosphate) were compared using a nonparametric Wilcoxon Signed-rank test, since the nutrients were collected at two time points and the data violated the assumptions of normality even after log-transformation.

The temperature and light logger data from Namena and Cousteau were combined into monthly measurements. Since the two replicate loggers at each site were not significantly different (p > 0.05), these data were averaged for Namena and Cousteau sites, respectively. When the data were downloaded, the light readings were not reliable after four months due to biofouling, hence the data were filtered to the first four months of reliable data. The monthly average temperature and light conditions at Namena were compared to Cousteau using Welch’s t tests following Shapiro Wilks tests for normal distribution of the data. Two measures of light conditions were analyzed: the total incident light and average light conditions monthly at each site.

All statistical analyses were conducted using R Studio v3.0.1^94^.

The datasets generated during and/or analyzed during the current study are available from the corresponding author on reasonable request.
